# Silanized Silica-Encapsulated Calcium Carbonate@Natural Rubber Composites Prepared by One-Pot Reaction

**DOI:** 10.3390/polym12112668

**Published:** 2020-11-12

**Authors:** Yao Yu, Junyi Zhang, Hongzhen Wang, Zhenxiang Xin

**Affiliations:** Key Laboratory of Rubber-Plastics, Ministry of Education, Shandong Provincial Key Laboratory of Rubber-Plastics, School of Polymer Science and Engineering, Qingdao University of Science and Technology, Qingdao 266042, China; 2018020073@mails.qust.edu.cn (Y.Y.); 4019020067@mails.qust.edu.cn (J.Z.); xzx@qust.edu.cn (Z.X.)

**Keywords:** calcium carbonate, silica, surface modification, wet mixing

## Abstract

This article demonstrates the one-pot reaction, an efficient and environmentally friendly organic synthesis method, utilized to prepare the silanized silica-encapsulated calcium carbonate@natural rubber composites (SSC@NR), following first mixing the calcium carbonate (CaCO_3_) solution, silica (SiO_2_) sol solution and a small amount of Si-69 solution, to modify the surface of CaCO_3_ particles, and then wet mixing with natural rubber latex. The obtained silanized silica-encapsulated calcium carbonate (SSC) particles were tested by TGA, FTIR and XRD, to substantiate the effect of surface modification. Moreover, the effects of the amount of SSC on the Mooney viscosity, curing characteristics, physical and mechanical properties and dynamic mechanical properties of the SSC@NR were investigated. The results show that the surface of modified CaCO_3_ is effectively coated with SiO_2_ particles by means of physical and chemical combination, to achieve the effect of surface coating. When the optimum amount of SSC filler is 40 phr, the SSC can form better physical adsorption and chemical combination with the NR molecular chains and can be evenly dispersed in the rubber matrix, resulting in the conspicuous improvement of physical and mechanical properties, such as the tensile strength, tear strength, elongation at break and abrasion resistance. Meanwhile, the compound with SSC has preferable processability and dynamic mechanical properties.

## 1. Introduction

Calcium carbonate (CaCO_3_) is a kind of widely used inorganic chemical raw material. In recent years, due to the rapid development of rubber industry, plastic industry, coating industry, building materials and paper industry, the development of calcium carbonate industry has been promoted correspondingly [1,2,3,4]. The main preparation methods of CaCO_3_ are as follows: double decomposition, carbonation and hypergravity carbonation [5,6,7,8]. The CaCO_3_ is an important inorganic filler which can be used for reinforcement, due to the advantages of low cost, less pollution, good mixing processability and good reinforcement effect. As we all know, filler reinforcement is an important way to improve the mechanical properties of rubber. It is achieved by forming a kind of interphase which is combined between filler and rubber, which requires the filler to achieve uniform dispersion in the rubber matrix [9,10]. However, CaCO_3_ particles are very easy to aggregate, owing to their small particle size, large specific surface area and high surface free energy [11]. In addition, the surface of CaCO_3_ particles has hydroxyl groups that exhibit polarity and have obvious characteristics of hydrophilicity and oleophobicity, making it difficult to disperse uniformly in the organic matrix. Therefore, during the process of blending CaCO_3_ particles with rubber, plastic and other materials, it is easy to form surface defects instead of chemical crosslinking, which reduces the comprehensive properties of the composites [12].

The surface modification of CaCO_3_ particles is the key to solve the problem of poor compatibility between CaCO_3_ and organic materials. Surface modification methods of CaCO_3_ particles include the following: local chemical reaction modification, surface chemical coating modification, high-energy surface modification and mechanochemical modification [13,14,15,16]. The surface of modified CaCO_3_ particles has better affinity to organic substrates and new features of hydrophobicity, so as to improve the dispersion of CaCO_3_ particles and interface compatibility with the organic matrix [17,18]. Jiang et al. [19] pretreated ground calcium carbonate (GCC) particles with NaOH, and then modified them with aminopropyltrimethoxysilane (APS) and used them to fill PVC. The results showed that the dispersion of fillers in the PVC matrix was improved by surface modification. Especially the use of GCC-OH-APS improves the tensile strength and impact strength of the composite; Zhou et al. [20] modified nano-CaCO_3_ with methacrylic acid (MAA) in situ and used it to reinforce EPDM. The experiment shows that MAA can form an ionic bond between the surface of CaCO_3_ particles and the matrix, thus enhancing the interaction between them and improving the tensile strength and modulus of vulcanizate. Poompradub et al. [21] modified the surface of CaCO_3_ nanoparticles with gallic acid. The experimental results indicate that gallic acid and the surface of CaCO_3_ nanoparticles formed an effective combination, and modified CaCO_3_ particles have excellent antioxidant properties. The mechanical properties of NR vulcanizates filled with gallic acid modified CaCO_3_ improved with the increase of loading. The modification of CaCO_3_ is mainly focused on organic modification. The organic modifier is adsorbed on the surface of CaCO_3_ particles by physical or chemical action. However, there are few reactive sites on the surface of CaCO_3_ particles, making this acting force not intense.

Silica (SiO_2_), as a widely used filler, has excellent acid- and heat-resistance, electrical insulation and reinforcing properties. The SiO_2_ particle has a large number of siloxane groups on its surface and has high surface activity. Its ultrafine effect can effectively improve the comprehensive properties of composite materials [22,23,24]. Therefore, the composites reinforced with SiO_2_ have attracted much research attention in the last few decades, attributed to the superior properties of SiO_2_ nanoparticles [25,26]. However, the SiO_2_ particle also has certain defects; its surface contains a large amount of silicon hydroxyl, which shows strong hydrophilicity and makes it easy to agglomerate. Therefore, its poor dispersion in the organic matrix and poor compatibility with non-polar matrix lower the processability and physical properties of the composites [27,28]. In order to enhance the interaction and interfacial compatibility between SiO_2_ and the polymer, the surface of SiO_2_ was functionalized. Functionalized SiO_2_ can endow inorganic components with reactivity and polymerizability to form covalent crosslinking with polymers [29,30]. Silane treatment of SiO_2_ particles with coupling agent is an effective method of functionalization. After hydrolysis, silane coupling agent can react with hydroxyl groups on SiO_2_ particles, to eliminate hydrophilic groups, thus contributing to excellent dispersions and interfacial interactions between the surface-modified SiO_2_ and the polymers [31,32,33].

The silica-encapsulated calcium carbonate (SC), a class of composite particles with core–shell structure, are prepared by coating a layer of SiO_2_ film on the surface of CaCO_3_ particles, which can integrate their advantages [34,35]. The composite particles endow part of the properties of SiO_2_ to the CaCO_3_ particles, which improves its surface properties and surface reaction activity, reinforcing performance and simultaneously reducing the production cost. The preparation methods of SC are mainly mechanical method and sol–gel method [11,36,37]. For example, Jiang et al. [38] added triethanolamine into ethanol solution and added CaCO_3_ solution under intense stirring. Then tetraethyl orthosilicate was added slowly in a 40 °C water bath. After stirring for 30 h, nano-CaCO_3_/SiO_2_ core–shell composite was prepared; Zhang et al. [39] dripped sodium silicate solution when the calcium hydroxide slurry was carbonized to pH 9.0, controlled the rate of carbon dioxide, stopped the reaction when the pH value of the system dropped to 7.0, and then aged for 2 h to prepare nano-CaCO_3_/SiO_2_ composite particles. Cui et al. [40] successfully prepared a new type of CaCO_3_-SiO_2_ composite particles with core–shell structure by mechanochemical method and applied it to reinforce silicone rubber. The results show that SiO_2_ particles can be uniformly fixed on the surface of CaCO_3_. The CaCO_3_-SiO_2_ composite particles can be well dispersed in silicone rubber, which not only reduces the use of SiO_2_, but also significantly improves the mechanical properties of silicone rubber. Nano-CaCO_3_/SiO_2_ composite particles with core–shell structure can be used in coating, papermaking, plastics, rubber and other industries, and the research in rubber field is very extensive.

Inspired by the above facts, this experiment adopts a one-pot reaction to coat ground CaCO_3_ particles with SiO_2_ particles, and carries out surface silanization treatment by adding Si-69 coupling agent. Then the as-fabricated silanized silica-encapsulated calcium carbonate (SSC) solution was poured into natural rubber latex, for wet mixing, to prepare silanized silica-encapsulated calcium carbonate@natural rubber composites (SSC@NR). Compared with other reports on the modification of CaCO_3_ particles [17,19,38], the one-pot reaction can be used to obtain particles with complex structure directly from relatively simple and easily available raw materials, without separation of intermediates, which has the characteristics of simplicity, high efficiency and low cost. The raw materials we selected can be easily obtained in industrial production. During the experiment, water was used as solvent, and no toxic organic reagent was used. Especially for the selected neutral silica sol solution, the thermal motion of the sol particles will aggravate the Brownian motion at a relatively mild temperature (80 °C), which will increase the collision probability between particles, resulting in the instability of sol system and the coagulation of SiO_2_ particles on the surface of CaCO_3_ particles to form a dense silica coating. Therefore, this experimental method also has the characteristics of low energy consumption and environmental friendliness, and it provides the feasibility for practical production in industry.

## 2. Experimental

### 2.1. Materials

The natural rubber latex (30% rubber content) was produced by Hainan Natural Rubber Industry Group Co., Ltd. (Haikou, China). Si-69, calcium carbonate solution and neutral silica sol were provided by Rhein Chemie Co., Ltd. (Qingdao, China). Anhydrous calcium chloride was analytical grade and obtained from Sinopharm Chemical Reagent Co., Ltd. (Beijing, China). Zinc oxide (ZnO), stearic acid (SA), *N*-isopropyl-*N*′-phenyl-4-phenylenediamin (4010NA), 2,2′-dibenzothiazoledisulfde (DM), 1,3-diphenylguanidine (D) and sulfur (S) were all industrial-grade and kindly supplied by Sanlux Co., Ltd. (Shaoxing, China).

### 2.2. Preparation of the SSC@NR

**Figure 1:**
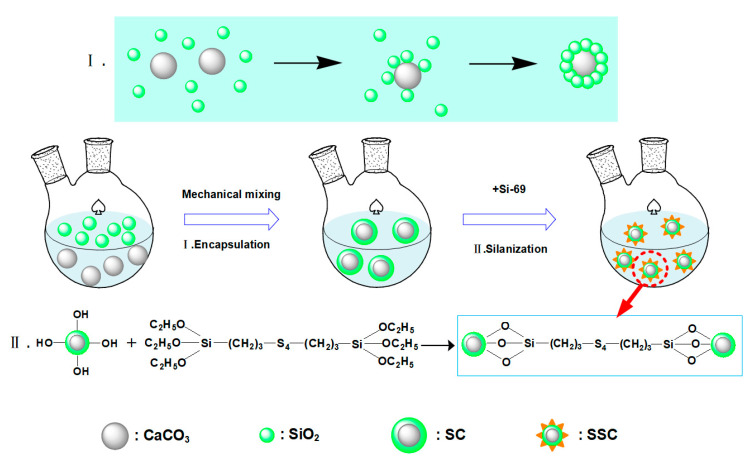
Schematic diagram for the preparation process and mechanism of silanized silica-encapsulated calcium carbonate (SSC)**.**

The 30% calcium carbonate solution (ground calcium carbonate) was poured into a flask, together with neutral silica sol solution, and mechanically stirred in a constant-temperature water bath, at 80 °C. The mass ratio of silica sol relative to CaCO_3_ is 1:8. Then, slowly, a small amount of Si-69 solution was added during the stirring process. After 3 h of reaction, the SSC solution was prepared. The as-fabricated SSC solution was centrifuged, filtered and washed with absolute ethanol, three times. The white powder was dried in a vacuum oven, at 40 °C, for 12 h, and the chemical structure and microstructure were tested. The schematic diagram for the preparation process and mechanism of SSC is shown in Figure 1.

The natural rubber latex with solid content of 30% was added into the reactor and mechanically stirred with the obtained SSC solution, at room temperature, for 0.5 h. After mixing evenly, we used 5% calcium chloride (CaCl_2_) solution for demulsification. The rubber block was crushed and granulated, and the calcium chloride was removed by soaking in water for about 5 h. Finally, the rubber particles were put into a vacuum oven and dried at 105 °C, for 4 h, and the SSC@NR composites were prepared.

The composites were plasticized on the double-roll open mill for several times. After the raw rubber was wrapped, zinc oxide (5 phr), stearic acid (2 phr), antioxidant 4010NA (1 phr), accelerator DM (1.2 phr), accelerator D (0.6 phr) and sulfur (2 phr) were added, in batches, for mixing. The compounds were prepared after thinning, making triangular package 5 times and slicing. The compounds had to be put aside for 24 h, and a piece of compound which is approximately round was cut and placed in a cure rheometer, to measure the curing characteristics at 160 °C. According to the optimum cure time T_90_, the vulcanizates were obtained by curing the compounds with a flat-panel curing press.

### 2.3. Experimental Variables

To examine the effect of the silanization and silica-encapsulated calcium carbonate content on the curing characteristics, processing properties and mechanical properties of the SSC@NR, the experimental variables are set as shown in Table 1.

### 2.4. Characterization and Testing

The SiO_2_, CaCO_3_ and SSC_3h_ were characterized by Fourier transform infrared spectrometer (FTIR; VERTEX 70, Bruker Optik GmbH Co., Ettlingen, Germany), using the absorption mode under a wave ranging from 4000 to 400 cm^−1^, with a resolution of 4 cm^−1^. The samples were pressed into pellets, together with potassium bromide.

X-ray diffraction (XRD) pattern of the CaCO_3_ and SSC_3h_ was recorded on an X-ray diffractometer (D-MAX2500-PC, Rigaku Corporation, Tokyo, Japan). The scanning rate was 5°/min, and the test angle was 5°–60°.

Thermogravimetric analysis (TGA) of the SiO_2_, CaCO_3_, SSC_1h_ and SSC_3h_ was performed on TGA-Instruments (209 F1, NETZSCH-Gerätebau GmbH, Selb, Germany), under nitrogen atmosphere. The samples for TGA tests were heated at a heating rate of 20 °C/min and temperature range of 60 to 900 °C.

The Mooney viscosity (ML_(1+4)100 °C_) of the CaCO_3_@NR and SSC@NR compounds was determined by a Mooney viscometer (GT-7080S2, GOTECH Testing machines Co., Ltd., Taiwan, China), at 100 °C, after 1 min preheating and 4 min running time.

The dynamic mechanical performances of the CaCO_3_@NR and SSC@NR compounds were analyzed, using RPA2000 (Alpha Technologies Co., Ltd., Akron, OH, USA), at 60 °C. For the rubber compounds, the strain amplitude was varied from 0.2% to 200%, at the test frequency of 1.67 Hz.

The CaCO_3_@NR and SSC@NR compounds which are approximately round were cut and placed in a cure rheometer (GT-M2000-A, GOTECH Testing machines Co., Ltd., Taiwan, China), to measure the curing characteristics, at 160 °C.

The mechanical properties of the CaCO_3_@NR and SSC@NR composites were investigated by using a universal testing machine (Z005, Zwick/Roell GmbH Co., Ulm, Germany), in accordance with ISO 528:2009, at a crosshead speed of 500 mm/min. 

Schopper (DIN) abrasion test of the CaCO_3_@NR and SSC@NR composites was carried out at room temperature, using a DIN abrasion tester (GT-7012-D, GOTECH Testing machines Co., Ltd., Taiwan, China). The cylindrical rubber sample was pressed on a rotating roller wrapped with sandpaper with 10N contact pressure. When the rotation speed of the roller is 40 r/min, the sample was moved horizontally on the roller, and the abrasion volume of the rubber sample after 40m stroke was measured.

## 3. Results and Discussion

### 3.1. The Chemical Structure and Microstructure of SSC

**Figure 2:**
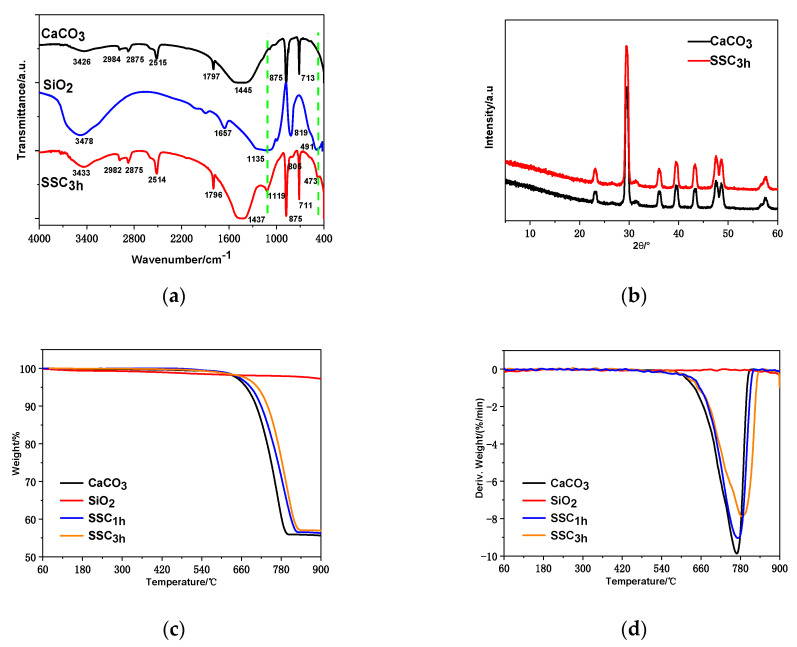
(**a**) FTIR spectrum of SiO_2_, CaCO_3_ and SSC_3h_; (**b**) XRD spectra of CaCO_3_ and SSC_3h_; (**c**) TGA curves of SiO_2_, CaCO_3_, SSC_1h_ and SSC_3h_; and (**d**) DTGA curves of SiO_2_, CaCO_3_, SSC_1h_ and SSC_3h_.

The SiO_2_, CaCO_3_ and SSC_3h_ were tested and analyzed by infrared spectrometer, respectively, and their infrared spectra were drawn on the same diagram, as shown in Figure 2a, for comparison. The main absorption peaks of CaCO_3_ particles were 713, 875 and 1445 cm^−1^, respectively. The absorption peak of 3426 cm^−1^ is the absorption peak of crystal water, indicating that CaCO_3_ particles contain water molecules; the absorption peak of 1445 cm^−1^ is caused by the symmetric stretching vibration of C–O bond; the absorption peak of 875 cm^−1^ is caused by the antisymmetric stretching vibration of C–O bond; and the absorption peak of 713 cm^−1^ is caused by the deformation vibration of C–O bond. These characteristic peaks show that CaCO_3_ particles have a calcite structure (standard CaCO_3_ infrared spectra) [41]. The main characteristic absorption peaks of SiO_2_ are 1629, 1135, 819 and 491 cm^−1^, which are caused by bending vibration of H–O bond, antisymmetric stretching vibration of Si–O bond, symmetric stretching vibration of Si–O bond and bending vibration of Si–O bond, respectively [42]. The infrared spectrum of SSC not only keeps the original characteristic absorption peaks of the CaCO_3_ particles, but also shows the antisymmetric stretching vibration peak and bending vibration peak of Si–O–Si, which are located at 1119 and 473 cm^−1^, respectively, indicating that the surface of modified CaCO_3_ particles is successfully coated with SiO_2_ particles [34]. Compared with unmodified CaCO_3_ particles, the absorption peak of SSC at 1437 cm^−1^ is narrowed, and the absorption peak at 1119 cm^−1^ is red-shifted, which shows that the combination of CaCO_3_ and SiO_2_ is not only in physical way, but also in the formation of chemical bond. In addition, the absorption peak of SSC at 3433 cm^−1^ indicates that there is bound water on its surface, but compared with CaCO_3_, the absorption peak is stronger, indicating that the surface of SiO_2_ also contains bound water.

The X-ray diffraction spectra of the CaCO_3_ and SSC_3h_ are shown in the Figure 2b. The positions of diffraction peaks of the SSC_3h_ are 23.2°, 29.4°, 36.0°, 39.5°, 43.3°, 47.5°, 48.7°, 56.6° and 57.7° respectively, which are basically the same as those of CaCO_3_ particles. These diffraction peaks are characteristic peaks of calcite. In addition, there is almost no difference in the peak strength and the interplanar spacing of the diffraction peaks of two kinds of particles. It demonstrates that the surface of surface-modified CaCO_3_ particles is successfully coated with SiO_2_ particles. The SiO_2_ in the passive layer is in an amorphous state and does not change the crystal structure of CaCO_3_. This is consistent with the XRD test results of SiO_2_-coated CaCO_3_ in the experiment of Lu et al. [35].

SiO_2_, CaCO_3_ and SSC with surface-modification time of 1 h (SSC_1h_) and 3 h (SSC_3h_) were analyzed and tested by using a thermogravimetric analyzer, and their TGA curves were drawn on the same diagram, for comparison. The TGA and DTGA curves of samples are shown in Figure 2c,d, respectively. As shown, the TGA curve of SiO_2_ decreases slowly with increasing temperature, indicating that there is bound water in SiO_2_, but no decomposition of SiO_2_ occurs within this temperature range. CaCO_3_ particles begin to decompose at a temperature of 620 °C, which is the initial decomposition temperature (*T*_0_), generating CaO and CO_2_. However, the *T*_0_ of the SSC_1h_ is higher than that of CaCO_3_ particles, indicating that the heat-resistance of the modified ones is improved. In addition, the degradation rate (C_f_) of thermal degradation reaction at temperature (*T*_f_) of the SSC_1h_ is smaller than that of CaCO_3_ particles, indicating that the surface-modified CaCO_3_ particles are effectively coated with SiO_2_ particles, forming the SSC and achieving a certain effect of surface modification. When the surface-modification time of CaCO_3_ particles is 3 h, its *T*_0_ is increased compared with the SSC_1h_, and its C_f_ is also reduced. It shows that, with the increase of the surface modification time, the heat-resistance of SSC is correspondingly improved, and the surface of CaCO_3_ particles is covered by more SiO_2_ particles, so the modification effect is better. The DTGA curves show the function relationship between the rate of change of mass with time and temperature. Figure 2d shows that the maximum degradation rate temperature (*T*_p_) of CaCO_3_ particles is 780 °C. When the temperature is higher than 780 °C, the decomposition rate slows down and eventually approaches zero, indicating that CaCO_3_ particles have been completely decomposed. However, the *T*_p_ of SSC_1h_ and SSC_3h_ increases in turn, while the maximum degradation rate decreases, in order. In the current temperature range, SiO_2_ does not decompose.

### 3.2. The Comprehensive Properties of SSC@NR Composites

#### 3.2.1. Mooney Viscosity of SSC@NR Compounds

**Figure 3:**
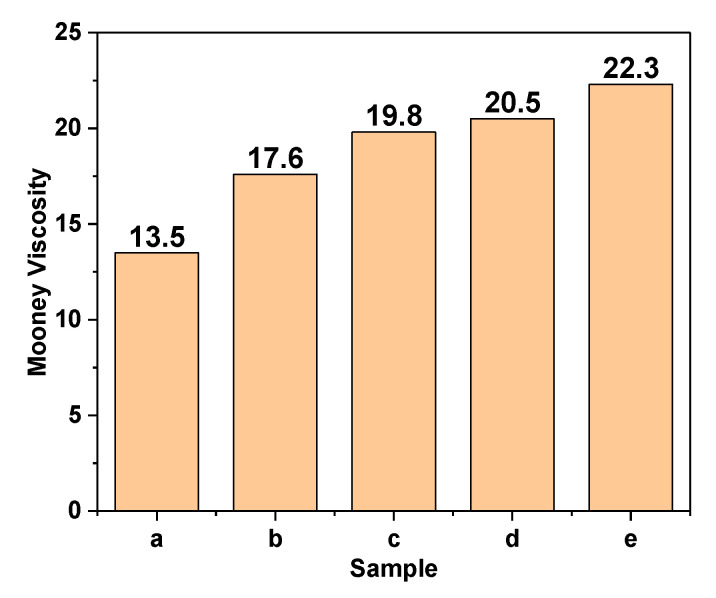
Mooney viscosity of SSC@NR compounds.

Mooney viscosity is regarded as an important indicator to measure the processing property of the composites. The Mooney viscosity of different samples is shown in the Figure 3. The SSC compared with unmodified CaCO_3_ particles significantly make the Mooney viscosity of the compounds increase by at least 30%, which demonstrates that the surface of the CaCO_3_ particles were successfully coated with SiO_2_ particles, which as the crosslinking point formed physical adsorption and chemical combination with the natural rubber molecular chains. Therefore, the increase of crosslinking density of the compounds led to the increase of Mooney viscosity. With the increase of the amount of SSC as fillers, the Mooney viscosity of the compounds increased correspondingly. It shows that more SSC particles were physically absorbed and chemically bonded to the natural rubber molecular chains, which can improve the crosslinking density of the compounds. Due to the existence of crosslinking points, it is not easy to cause relative slippage between the natural rubber molecular chains, resulting in an increase in the viscosity of the compounds, which makes the processing of compounds require greater external force and adversely affects the processing performance of compounds.

#### 3.2.2. Curing Characteristics of SSC@NR Composites

The curing characteristics of a series of compounds are shown in Table 2. It can be clearly concluded from the data that the SSC causes the ML value to improve, reflecting the decrease of the fluidity of the compounds, compared with compounds containing unmodified CaCO_3_ particles. Moreover, with the increase of the amount of SSC, the ML value increases gradually, which indicates that SSC can make the fluidity of the compounds worse and are not conducive to processing. MH-ML can be used to characterize the crosslinking density of composites, and the higher its value, the higher the crosslinking density. It can be seen that the crosslinking density of the compounds with SSC is higher than that of the compound with CaCO_3_ particles, and the crosslinking density of the compound improves gradually with the increase of the content of SSC. The increase of crosslinking density indicates that the number of crosslinking points between molecular chains increases correspondingly. Due to the hindered internal rotation of the single bond of the main chain near the crosslinking point, the compliance of the molecular chains of the composites is reduced, and slippage of the molecular chains is unlikely to occur. Therefore, SiO_2_ particles are effectively coated on the surface of the CaCO_3_ particles, which is similar to the crosslinking point to promote the formation of binding between the SSC and the natural rubber molecular chain. The scorch time (*T*_s1_) gives expression to the processing safety of rubber, and the larger the value, the higher the processing safety. It can be concluded from the table that the *T*_s1_ of the SSC_40_@NR is higher than that of the CaCO_3_@NR. Nevertheless, the scorch time of compounds decreases gradually with the increase of the content of SSC. The optimum cure time (*T*_90_) represents the curing rate of the compound. The values of *T*_90_ of the compounds raise with the increase of SSC, which is due to the delayed curing effect of SiO_2_ particles on compounds.

#### 3.2.3. Physical and Mechanical Properties of SSC@NR Composites

The physical and mechanical properties of a series of the SSC@NR vulcanizates tested at the room temperature (23 ± 2 °C) are shown in Table 3.

The tensile strength and elongation at break of the SSC_40_@NR vulcanizate are significantly improved compared with CaCO_3_@NR vulcanizate. This shows that, at a microscopic level, the compatibility and dispersion of the SSC in the rubber matrix is enhanced, and physical adsorption and chemical combination with the natural rubber molecular chains are effectively formed, which leads to an increase of the crosslinking points and crosslinking density. When the vulcanizate is deformed by an external force, it is less likely to produce relative slippage between the rubber molecular chains, which can well relax the external stress, so the reinforcing effect is achieved. However, the tensile strength and elongation at break of the vulcanizate show a downward trend with the increase of the content of SSC. This is because the continuous increase of the SSC causes excessive crosslinking density, resulting in the decrease of the molecular weight and the activity of the network chain between the crosslinking points. When the vulcanizate is deformed by the external force, it tends to form the stress concentration point and produce the microscopic cracks. The expansion of cracks leads to the macroscopic damage of the material. Meanwhile, too much of the fillers can easily cause agglomeration in the rubber matrix and lead to stress concentration, which makes the tensile properties of vulcanizate worse.

By comparing a series of data, it can be found that the tear strength of the SSC@NR vulcanizates are higher than that of the CaCO_3_@NR vulcanizate, and with the increase of its content, the tear strength performs an upward tendency. It is obviously indicated that the SSC can enhance the ability of the composites to resist the stress concentration around the notch and improve its toughness.

The hardness of the SSC@NR vulcanizates is significantly improved, which indicates that SSC enhances the rigidity of the compounds. The compression permanent deformation of the SSC_40_@NR vulcanizate is slightly lower than that of the CaCO_3_@NR vulcanizate, indicating that the SSC has better compatibility and dispersion with the rubber matrix. The increase of the number of crosslinking points in vulcanizate makes it difficult for the rubber molecular chain to move relatively when it is deformed by external force, and the vulcanizate can recover to approach the original shape when the external force is removed, which shows that the resilience of the material is improved. However, with the increase of the amount of SSC, the compression permanent deformation of vulcanizate increases accordingly, which is due to the agglomeration of too many filler particles. The agglomeration of filler particles can easily form stress concentration points. When the vulcanizate is deformed by external force, the crosslinked molecular chains are broken, the spatial crosslinking network is destroyed and the relative displacement between the molecular chains occurs. Even if the external force is removed, this part of the deformation cannot be elastically recovered, thus resulting in permanent deformation.

According to the analysis of the Schopper abrasion data of vulcanizates, it can be concluded that the abrasion resistance of SSC_40_@NR vulcanizate is improved compared to the CaCO_3_@NR vulcanizate, indicating that the dispersion of the SSC in rubber matrix is elevated and the interfacial binding force is better. However, with the increase of the content of the SSC, the abrasion resistance of the vulcanizate shows a downward trend. This may be due to the agglomeration of the filler, which results in stress concentration and the formation of crack sources, and the vulcanizate is prone to fatigue and fall off when subjected to repeated wear.

The comprehensive review of tensile strength and elongation at break of different polymer systems with various filler materials, compared with the present study as summarized in Table 4. 

Compared with other modified CaCO_3_/NR composites, our SSC@NR composites have higher tensile strength, which proves that the SiO_2_ coating layer can endow CaCO_3_ particles with more reactive points and enhance the crosslinking density of the composites. Compared with other coupling agent modified CaCO_3_/NR composites, the tensile strength of SSC@NR composites is equivalent, while the elongation at break has increased. Chen et al. [45] used sodium stearate and Si-69 to synergistically modify CaCO_3_/SiO_2_ and reinforces SBR. It can be seen clearly from the data that CaCO_3_/SiO_2_ has a better reinforcing effect than CaCO_3_. Cui et al. [40] filled the silicone rubber with CaCO_3_/SiO_2_ composite particles, and the tensile strength and elongation at break of the composites were also significantly improved. These are consistent with our research results. Therefore, encapsulating CaCO_3_ with SiO_2_ can give CaCO_3_ particles the excellent properties of SiO_2_, and the composites with natural rubber have more excellent mechanical properties. This research broadens the application range of CaCO_3_ and provides a new era in the reinforcement of NR.

#### 3.2.4. Dynamic Mechanical Properties of SSC@NR Composites

**Figure 4:**
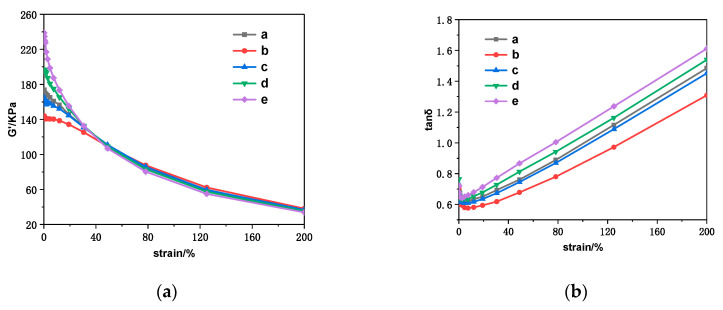
(**a**) Storage modulus (G’) and (**b**) loss factor (tan δ) versus strain for SSC@NR composites.

As shown in Figure 4a, which is the storage-modulus-strain curves of different specimens, the storage modulus (G’) of all compounds decreases with the increasing strain, which is defined as the Payne effect. The strength of the Payne effect manifests the dispersion of filler in rubber matrix, usually measured by the difference of G’ (∆G’) under low strain and high strain [46]. The smaller the ∆G’, the better the dispersion of the filler in the rubber matrix. The fillers in the natural rubber will spontaneously form a three-dimensional space network, which improves the G’ of composites. The increase of strain will lead to deformation or fracture of three-dimensional network of fillers, and the G’ creates a slight variation under small strain. However, when the strain is greater than 10%, the destruction rate of network increases rapidly, and G’ declines faster. This is because the molecular chains change from the initial orientation and slip to the gradual fracture, and the filler network failure is greater than the reconstruction, resulting in more damage to the network of fillers than reconstruction.

Comparing the curves of various compounds, it can be found that the Payne effect of the SSC_40_@NR compound is significantly lower than that of the CaCO_3_@NR compound, certifying that the compatibility and dispersion of the SSC in the rubber matrix are greatly improved and that they have more binding and adsorption with the natural rubber molecular chain. Nevertheless, with the increase of the content of SSC, the Payne effect has been strengthened, which indicates that too many fillers easily interact and form agglomeration, resulting in poor dispersion in the rubber matrix.

It can be observed from Figure 4b that the loss factor (tan δ) of all samples shows a trend of decreasing first and then improving with the increase of strain, and the increase rate of tan δ is accelerated after the strain is greater than 10%. This is because the movement of rubber molecular chains under small strain belongs to high elastic deformation, the hysteresis of composites is weak and its internal friction is small. When the strain reaches a certain value, the relative slip or fracture occurs between rubber molecular chains, and its friction with filler particles increases, leading to the increase of internal friction.

It can be found by comparing the tan δ of different samples under the same strain that the tan δ of the SSC@NR compound is significantly lower than that of the CaCO_3_@NR compound, and the tan δ also improves with the increase of the amount of SSC. The filler forms a physical adsorption and chemical combination with the rubber molecular chains. When the rubber molecular chains are subjected to dynamic action, the unevenly dispersed filler will agglomerate, and the relative sliding between the fillers and the molecular chains will increase, and more energy will be consumed by the destruction and reconstruction of the filler network.

## 4. Conclusions

In this research, through a facile, efficient and environmental one-pot reaction, silanized silica-encapsulated calcium carbonate (SSC) particles could be fabricated through the mixing of CaCO_3_ solution, neutral silica sol solution and Si-69 solution, and then wet-mixed SSC with natural rubber latex, to obtain SSC@NR composites. The characterization of FTIR, XRD and TGA confirmed that the surface of CaCO_3_ particles can be successfully encapsulated with SiO_2_ particles by physical and chemical methods. Compared with unmodified CaCO_3_, when the optimal amount of SSC is 40 phr, the dispersion of SSC in NR matrix is obviously enhanced, which could form better physical adsorption and chemical combination with the NR molecular chains. Therefore, the physical and mechanical properties of SSC_40_@NR composites, including tensile strength, tear strength, elongation at break and abrasion resistance, are significantly improved. Simultaneously, SSC_40_@NR composites have better dynamic mechanical properties. The preparation method outlined in this paper provides a significant potential for the surface modification of CaCO_3_. The prepared high-performance SSC@NR composites are expected to have practical and far-reaching applications in the rubber industry.

## Figures and Tables

**Table 1:** Experimental variables.

Samples	A ^1^	B ^2^	C ^3^	D ^4^	E ^5^
NR/phr	100	100	100	100	100
Calcium carbonate/phr	40	40	60	80	100
Silica sol/phr	-	5	7.5	10	12.5
Si-69/phr	-	2	3	4	5
Modification time/h	-	3	3	3	3

^1^ Composites with 40 phr unmodified CaCO_3_ (CaCO_3_@NR). ^2^ Composites with 40 phr SSC (SSC_40_@NR). ^3^ Composites with 60 phr SSC (SSC_60_@NR). ^4^ Composites with 80 phr SSC (SSC_80_@NR). ^5^ Composites with 100 phr SSC (SSC_100_@NR).

**Table 2:** Curing characteristics of SSC@NR compounds.

Project	Sample				
	a	b	c	d	e
ML/dN·m	0.356	0.533	0.873	0.892	1.010
MH/dN·m	22.091	23.061	29.920	33.195	37.334
MH-ML/dN·m	21.735	22.705	29.047	32.303	36.324
*T*_s1_/min	1.63	1.75	1.57	1.53	1.48
*T*_10_/min	1.80	1.93	1.80	1.83	1.83
*T*_90_/min	6.22	6.90	7.38	7.70	7.88
CRI/min^−1^	22.64	20.13	17.91	17.05	16.53

**Table 3:** Physical and mechanical properties of SSC@NR vulcanizates.

Property	Sample				
	a	b	c	d	e
Tensile strength/MPa	22.2 ± 1.1	25.6 ± 0.9	22.1 ± 1.2	20.2 ± 0.7	19.2 ± 1.0
Elongation at break/%	582 ± 38	629 ± 37	632 ± 41	556 ± 32	509 ± 35
100% Tensile modulus/MPa	1.2 ± 0.1	1.2 ± 0.1	1.8 ± 0.2	2.1 ± 0.1	2.8 ± 0.2
300% Tensile modulus/MPa	4.5 ± 0.2	4.3 ± 0.2	5.7 ± 0.4	6.9 ± 0.3	8.3 ± 0.4
Tear strength/N·mm^−1^	35.4 ± 1.5	38.1 ± 2.1	44.0 ± 1.4	46.3 ± 1.9	46.8 ± 1.5
Shore A hardness	44 ± 1	46 ± 1	55 ± 1	61 ± 1	64 ± 1
Compression permanent distortion/%	3.5 ± 0.1	3.3 ± 0.2	4.1 ± 0.1	4.9 ± 0.2	5.7 ± 0.3
Schopper abrasion/cm^3^	0.173 ± 0.012	0.163 ± 0.009	0.174 ± 0.010	0.177 ± 0.007	0.189 ± 0.013

**Table 4:** Comprehensive analysis of various composites and its tensile strength and elongation at break.

Polymer Matrix	Filler Materials	Tensile Strength/MPa	Elongation at Break/%	Reference
NR	20 phr CaCO_3_	20.1	713	[21]
NR	5 phr WESNCC/MNR	20.7	670	[43]
NR	70 phr SiO_2_	26.2	575	[33]
NR	50 phr SiO_2_	15.1	555	[44]
SBR	75 phr CaCO_3_/SiO_2_ 75 phr CaCO_3_	14.1 8.1	1036 1291	[45]
Silicone rubber	45 phr CaCO_3_- SiO_2_ 45 phr CaCO_3_	1.0 0.4	157 99	[40]
NR	40 phr SSC 40 phr CaCO_3_	25.6 22.2	629 582	Our work
